# ΔNp63 regulates the expression of hyaluronic acid-related genes in breast cancer cells

**DOI:** 10.1038/s41389-018-0073-3

**Published:** 2018-08-24

**Authors:** Veronica Gatti, Claudia Fierro, Mirco Compagnone, Federica Giangrazi, Elke Katrin Markert, Lucilla Bongiorno-Borbone, Gerry Melino, Angelo Peschiaroli

**Affiliations:** 1 National Research Council of Italy, (CNR), Institute of Cell Biology and Neurobiology (IBCN), CNR, Monterotondo, Rome, Italy; 2 Department of Experimental Medicine and Surgery, University of Rome “Tor Vergata”, Via Montpellier 1, 00133 Rome, Italy; 3 Institute of Cancer Sciences, University of Glasgow, Garscube Estate, Switchback Road, Glasgow, G61 1BD UK; 4 Medical Research Council, Toxicology Unit, Hodgkin Building, Leicester University, Lancaster Road, P.O. Box 138, Leicester, LE1 9HN UK; 5 National Research Council of Italy, (CNR), Institute of Translational Pharmacology (IFT), Via Fosso del Cavaliere 100, Rome, 00133 Italy; 6 Present Address: Paediatric Haematology/Oncology Department, Bambino Gesù Children’s Hospital IRCCS, Piazza S. Onofrio 4, 00165 Rome, Italy; 7 Present Address: Molecular Cell Biology Laboratory, Department of Genetics, The Smurfit Institute, Trinity College, The University of Dublin, Dublin 2, Ireland

## Abstract

Triple negative breast cancers (TNBC) represent the most aggressive and clinically relevant breast carcinomas. On the basis of specific molecular signature, the majority of TNBC can be classified as basal-like breast carcinoma. Here, we report data showing that in basal-like breast carcinoma cells ΔNp63 is capable of sustaining the production of the hyaluronic acid (HA), one of the major component of the extracellular matrix (ECM). At molecular level, we found that ΔNp63 regulates the expression of HA-related genes, such as the HA synthase HAS3, the hyaluronidase HYAL-1 and CD44, the major HA cell membrane receptor. By controlling this pathway, ∆Np63 contributes to maintain the self-renewal of breast cancer stem cells. Importantly, high HAS3 expression is a negative prognostic factor of TNBC patients. Our data suggest that in basal-type breast carcinoma ∆Np63 might favor a HA-rich microenviroment, which can sustain tumor proliferation and stemness.

## Introduction

Breast tumors are one of the most heterogeneous human cancers and different types have been categorized on the basis of histological and molecular features^1^. Triple negative breast cancers (TNBC), which represent 15% of breast carcinomas, are defined by the lack of *HER2* gene amplification and the absence of estrogen and progesterone receptors^2^. From a clinical point of view, TNBC are refractory to targeted therapies, and the only therapeutic option is the conventional chemotherapy-based approach. On the basis of specific molecular profile, TNBC can be further divided into sub-types, among which the basal-like breast carcinomas represent the majority of TNBC^3–5^. ΔNp63 isoforms (herein refereed as ΔNp63) are N-terminal truncated variants of the transcription factor p63 whose expression and activity has been functionally associated with the basal-like breast phenotype. Albeit lacking a canonical transcriptional activation domain, ΔNp63 is able to transcriptionally activate several transcriptional programs involved in a variety of tumor-related pathways^6–18^. In particular, in luminal and basal-breast carcinoma ΔNp63 acts as a key regulator of the tumor cell stemness as loss of ΔNp63 reduces the self-renewal ability of cancer progenitors and delays tumor growth after their transplantation^19,20^. Moreover, ΔNp63 augments the percentage of stem cell-like sub-populations in breast carcinoma cell lines^21^, reinforcing the concept that ΔNp63 is an important regulator of the stemness properties of breast cancer cells, a feature strictly correlated with the tumor aggressiveness. In line with these evidences, ΔNp63 positively regulates the invasion and migration of breast tumor cells^22^. In addition to act as a transcriptional activator, ΔNp63 is also able to repress the expression of several genes by different mechanisms^23–25^.

During tumor progression, the extracellular matrix (ECM) undergoes extensive remodeling in order to sustain the invasive and proliferative capabilities of tumor cells^26–29^. One of the major component of the ECM is hyaluronic acid (HA), a non-sulfated, linear glycosaminoglycan (GAG), which not only contributes to tissue architecture and hydration but also provides a favorable microenvironment for cell proliferation and migration^30–32^. Accordingly, HA is produced at higher level in the growing fetal tissues and during embryo development it supports the proliferation and migration of the stem cells^33^. However, the response of the cells to a HA-rich ECM depends not only on the amount of HA but also on the size of the GAG chains, and the presence of specific cell-surface receptors such as CD44^34–36^. HA metabolism is finely regulated by the opposite functions of two classes of enzymes: the HA synthases and the hyaluronidases^37^. The HA synthases catalyze the synthesis of HA on the plasma membrane and three mammalian isoenzymes (HAS1, HAS2, and HAS3) are present in the human genome. These enzymes display distinct catalytic properties in terms of size of HA synthesized^37,38^. HA synthesis is counterbalanced by a degradative pathway that clears HA by endocytic uptake and/or HA hydrolysis^39^. Among the six human hyaluronidase (*HYAL*) genes (*HYAL-1-4, HYAL-P1*, and the sperm-specific *PH-20*), H*YAL-1* and *HYAL-2* are the best characterized.

In several pathological conditions, including tumor development, HA metabolism and signaling are commonly deregulated^30^. During tumor progression, deregulation of HA metabolism is often associated with alterations of the enzymes that regulate HA synthesis and degradation. Overexpression of either HAS2 or HAS3 is associated with higher malignancy or metastasis in several tumor types, such as breast, prostate, and colon carcinomas^40–45^. We have previously demonstrated that in head and neck squamous cell carcinoma (HNSCC) ΔNp63 controls the expression of the HA-related genes *HAS3*, *HYAL1*, and *CD44*^46^. Here, we analyze the role of the ΔNp63-dependent regulation of HA metabolism in basal breast carcinoma reporting that the ΔNp63-HA metabolism axis might be important to regulate the stemness properties of basal breast carcinoma.

## Results and discussion

### ΔNp63 regulates the expression of HA metabolism genes in breast cancer cells

**Fig. 1:**
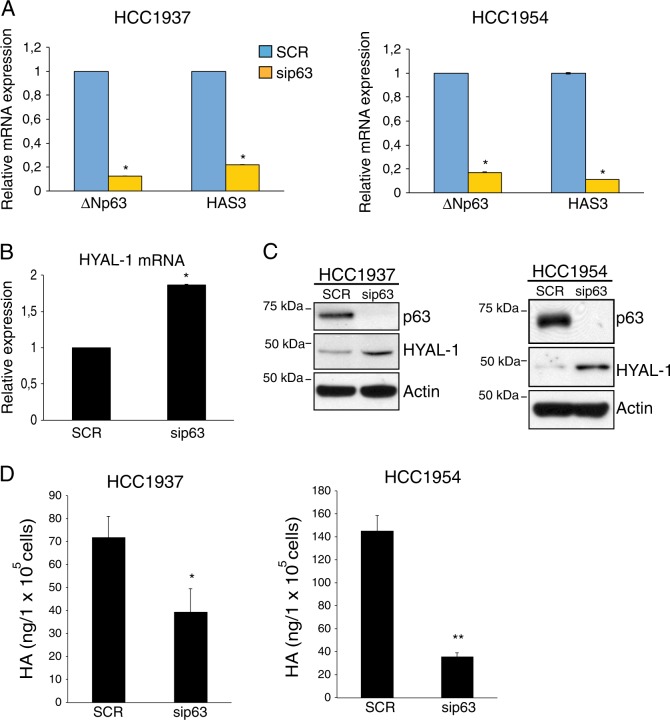
p63 sustains the HA metabolism by controlling the expression of the HA-related genes HAS3 and HYAL-1. **a** HCC1954 and HCC1937 breast carcinoma cell lines were grown in RPMI-1640 medium (Gibco, Invitrogen); H1299 cells (non-smal cell lung cancer cell line) were cultured in Dulbecco’s modified Eagle’s medium (Gibco, Invitrogen) supplemented with 10% v/v fetal bovine serum (FBS), 100 µg/mL penicillin and 100 µg/mL streptomycin (Gibco, Invitrogen). Cells were cultured at 37 °C with 5% CO_2_. Breast carcinoma cell lines were purchased from ATCC and routinely tested for mycoplasma contaminations. HAS3 and ΔNp63 mRNA levels were quantified by Real-Time PCR analysis (qRT-PCR) in the indicated basal-type breast tumor cell lines transfected with scrambled (SCR) or p63 siRNA (sip63) oligos. For siRNA oligos transfection cells were seeded at a density of 1.4 × 10^5^ cells/well in a six-well plate and transfected with oligos using RNAimax (Invitrogen) according to manufacturer’s instructions. Smart pool siRNA oligos direct against p63, HAS3 mRNA, and non-relevant gene (scramble) were purchased by Dharmacon (Thermo Scientific). Cells were collected 48 h after transfection and lysates were subjected to qRT-PCR analysis. Total mRNA was isolated using the RNeasy mini kit (Qiagen, Duesseldorf, Gemrany) following the manufacturer’s recommendations. Total RNA was quantified using a NanoDrop Spectophotometer (Thermo Scientific, Delaware, USA) and used for cDNA synthesis using Superscript Reverse Transcriptase (Promega, Fithburg, WI, USA), according to the manufacturer’s protocol. cDNA was subsequently used for qRT-PCR. Each 25 µl reaction contained 2X SYBR-Green PCR Master Mix (Promega), 2 µl cDNA and the appropriate specific primers (0.5 µM). Amplification and fluorescence detection according to the manufacturer’s instructions was performed using the ABI PRISM 7700 Sequence Detection System (Applied Biosystems, France). The expression of each gene was defined from threshold cycle (C_t_), and the relative expression levels were calculated using the 2-ΔΔC_t_ method. The primers used for qRT-PCR are the following: human ΔNp63 for 5′-GAAGAAAGGACAGCAGCATTG-3′; rev 5′-GGGACTGGTGGACGAGGAG-3′; human HAS3 for 5′-TGTGCATTGCCGCATACC-3′; rev 5′-CCGAGCGCAGGCACTT-3′; human GAPDH for 5′-AGCCACATCGCTCAGACA-3′; rev 5′-GCCCAATACGACCAAATC-3′; human Actin for 5′-GTTGCTATCCAGGCTGTG-3′; rev 5′-AATGTCACGCACGATTTCCCG-3′; Bars represent the mean of three technical replicates (*n* = 3, PCR runs) ± SD and are representative of three independent experiments (*n* = 3 biological replicates). No data were excluded form the analysis. *p- value < 0.05. **b** HYAL-1 mRNA levels were measured by qRT-PCR in HCC1937 cells transfected as in **a**. The following oligos were utilized for HYAL-1 mRNA expression analysis: human HYAL-1 for 5′-CGATATGGCCCAAGGTTTAG-3′; rev 5′-ACCACATCGAAGACACTGACAT-3′. Bars represent the mean of three technical replicates (*n* = 3, PCR runs) ± SD and are representative of two independent experiments (*n* = 2 biological replicates). No data were excluded form the analysis. *p- value < 0.05. **c** Total protein lysates extracted by the HCC1937 and HCC1954 cells transfected as in **a** were analyzed by immunoblotting (IB) using antibodies to the indicated proteins. IB was performed as previously described^68.^ The following antibodies were utilized: rabbit monoclonal anti p63-α D2K8X (Cell Signaling Technology); mouse monoclonal anti β-actin (AC-15) (Sigma-Aldrich) and rabbit polyclonal anti HYAL-1 (Sigma-Aldrich). **d** HCC1937 and HCC1954 cells (2 × 10^5^ cells/well) were transfected with scrambled (SCR) or p63 siRNA (sip63) oligos. Forty-eight hours after transfection growth medium of transfected cells was collected and extracellular hyaluronic acid (HA) levels were measured using Hyaluronan Enzyme-Linked Immunosorbent Assay Kit (HA-ELISA) (Echelon) following the manufacturer’s recommendations. The amount of hyaluronic acid (ng) was normalized per the number of cells (10^5^ cells). Bars represent the mean of four replicates (*n* = 3) ± SD. *p-value < 0.05; **p-value < 0.01. Statistical evaluation was determined by using a two-tailed *t*-test.

Several reports have demonstrated that ΔNp63-dependent signature expression is preferentially associated with the basal sub-type over the luminal subtype of TNBC^19^. Therefore, we tested whether the ΔNp63-dependent transcriptional regulation of HA-related genes previously observed in HNSCC occurs in this type of breast carcinoma. To this aim, we first analyzed the expression of the N-terminal p63 isoforms in two basal-type breast carcinoma cells, HCC1937 and HCC1954. By qRT-PCR analysis, we confirmed that ΔNp63 isoforms are the main p63 isoforms expressed in these breast cell lines (Figure S1A). To assess which ΔNp63 C-terminal splicing variants are expressed in the basal-type breast carcinoma cells, we performed an immunoblotting analysis and we found that the full-length ΔNp63α isoform is the only detectable ΔNp63 isoform expressed at protein level in these cells (Figure S1B). We then analyzed the effect of ΔNp63 silencing on the expression levels of HAS3. We observed that depletion of ΔNp63 efficiently decreases the mRNA levels of HAS3 (Fig. 1a). Interestingly, the expression of other HAS synthases, HAS1 and HAS2, is almost undetectable in basal-type breast carcinoma cell lines (Figure S1C).

By analyzing publically available p63 ChiP-seq data of basal breast tumor cell line cell MCFDIS, we found that p63 is able to occupy a p63-binding site (p63 BS) located in the *HAS3* promoter (Figure S1D), suggesting that ΔNp63 might directly regulates HAS3 expression. Parallel to the decrease of HAS3 expression we observed that ΔNp63 silencing increases the expression of the hyaluronidase gene *HYAL-1* at both mRNA and protein levels (Fig. 1b, c). In light of these findings, we tested whether ΔNp63 is capable of regulating HA levels. By ELISA assay we quantified HA levels in the growth medium of HCC1954 and HCC1937 cells upon depletion of ΔNp63 and we found that ΔNp63-depleted cells display a marked reduction of HA levels (Fig. 1d). These findings indicated that ΔNp63 is able to control the expression of HA metabolic genes, and, by doing that, stimulate HA production.

### ΔNp63 induces the expression of the HA receptor CD44 and favors EGF-R activation

**Fig. 2:**
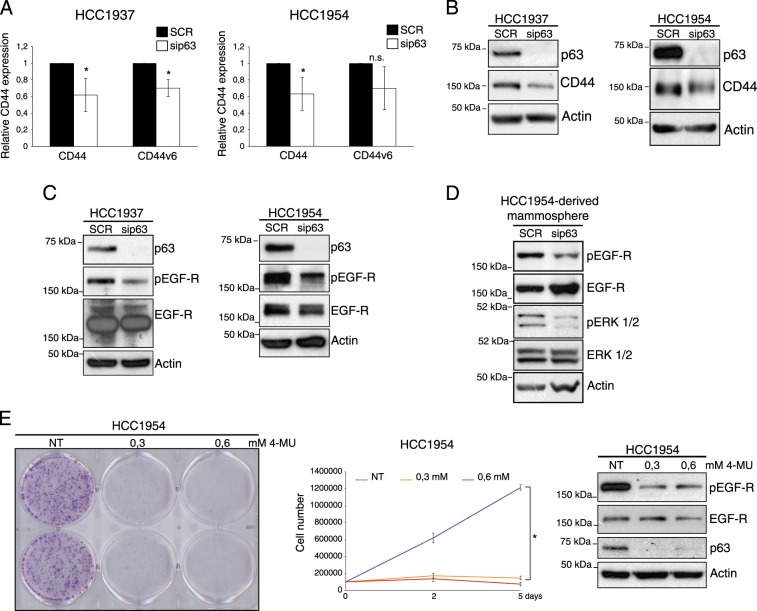
p63-dependent regulation of HA/CD44 pathway favors EGF-R activation **a** The indicated basal-type breast tumor cells transfected with scrambled (SCR) or p63 siRNA oligos (sip63) were analyzed for the expression of CD44 by qRT-PCR. We utilized the following CD44 oligos. CD44 total: forward 5′-CAACTCCATCTGTGCAGCAAA-3′; rev 5′-GTAACCTCCTGAAGTGCTGCTC-3′. CD44v6 forward 5′-AGTACAACGGAAGAAACAGCTA-3′; rev 5′-TGTCCCTGTTGTCGAATGG-3′. Bars represent the mean of four independent experiments (*n* = 4 biological replicates) ± SD. No data were excluded from the analysis. *p-value < 0.05. **b** The indicated basal-type breast tumor cells treated as in **a** were analyzed by immunoblotting using the antibodies for the indicated proteins. Mouse monoclonal antibody anti-CD44 (8E2) (Cell Signaling Technology) or mouse monoclonal anti-HCAM (CD44) (DF1485) (Santa Cruz Biotechnology) were utilized. **c** HCC1937 and HCC1954 cells were transfected as in **a** and total protein lysates were immunoblotted utilizing antibodies for the indicated proteins. Rabbit polyclonal anti-EGF Receptor (Cell Signaling Technology) and rabbit monoclonal anti-Phospho-EGF Receptor (Tyr1068) (D7A5) (Cell Signaling Technology) were utilized; **d** HCC1954 cells were transfected with scrambled (SCR), or p63 siRNA (sip63) oligos for 48 h and then transfected cells were plated for the sphere-forming assay. Briefly, breast cancer cells were plated in low-attachment 24-well culture plates at a density of 1000 cells per milliliter, in DMEM supplemented with 5 μg/mL insulin, 0.5 μg/mL hydrocortisone, 2% (vol/vol) B27 (Invitrogen), 20 ng/mL EGF and bFGF (BD Bio-sciences), and 4 μg/mL heparin (Sigma). The medium was made semisolid by the addition of 1% (vol/vol) methylcellulose to prevent cell aggregation. Total protein lysates were immunoblotted, utilizing antibodies for the indicated proteins. Rabbit polyclonal anti-Phospho-p44/42 MAPK (Erk1/2) (Thr202/Tyr204) (Cell Signaling Technology) and rabbit monoclonal p44/42 MAPK (Erk1/2) (Cell Signaling Technology, clone 137F5) were utilized. **e** Representative image of clonogenic assay (left panel) performed by treating HCC1954 cells (500 cell per well) with the indicated concentration of 4-methylumbelliferone (4-MU, Sigma-Aldrich) for 1 week. Growth curve (middle panel) was performed by treating 1 × 10^5^ cells with the indicated concentration of 4-MU for the indicated days. Cells were counted at the indicated time points in quadruplicates (*n* = 4 technical replicates). The mean ± SD at days 2 and 5 was calculated and plotted. The graph is representative of two independent experiments. *p-value < 0.05. In parallel, protein lysates were immunoblotted for the indicated proteins (right panel)

Many of the tumor-associated activities of HA are exerted by its interaction with the HA receptor CD44, which is an important player of key oncogenic signaling^34^. We have previously showed that ΔNp63 controls directly the expression of CD44 in HNSCC. To confirm this data in breast cancer cells, we analyzed CD44 expression in HCC1937 and HCC1954 cells upon p63 silencing. We found that in both cell lines ΔNp63 silencing decreases CD44 at mRNA and protein levels (Fig. 2a, b). Interestingly, in HCC1937 cells, p63 silencing decreases the expression of the CD44 splicing variant CD44v6, a key regulator of stem like properties of breast cancer stem cells^47^. By analyzing p63 ChIP-seq data of MCFDIS cells, we found several p63-binding sites, located in the *CD44* genomic locus (Figure S2), suggesting that ΔNp63 controls CD44 expression by physically binding to the *CD44* locus.

It has been shown that HA/CD44 interaction favors the activation of the epidermal growth factor receptor EGF-R^48–50.^ Therefore, we tested whether the ΔNp63-dependent regulation of the HA/CD44 axis is capable of promoting EGF-R activation in basal breast carcinoma. As shown in Fig. 2c, ΔNp63 silencing decreases EGF-R phosphorylation in HCC1954 and HCC1937 cells. Interestingly, EGF-R signaling is also negatively affected upon p63 silencing in HCC1954-derived mammosphere (Fig. 2d), reinforcing the concept that p63 and EGF-R signaling are important players of the cell stemness properties of breast tumor cells.^19,20,47,51^ Furthermore, treatment with 4-methylumbelliferone (4-MU), a chemical inhibitor of the enzymatic activity of the HAS synthases^52,53^ exerted a similar downregulation of EGF-R phosphorylation and a marked cytostatic effect (Fig. 2e). These data indicated that ΔNp63 is able to favor EGF-R activation by likely modulating HA metabolism and signaling.

### HA/CD44 axis sustains breast tumor cell stemness

**Fig. 3:**
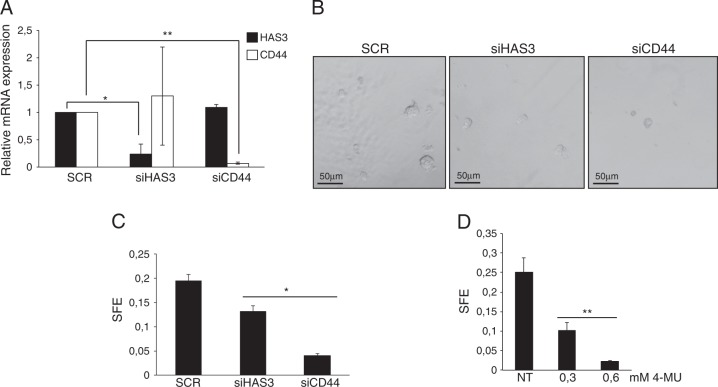
HA/CD44 pathway regulates breast tumor stemness. **a** HCC1954 cells were transfected with scrambled (SCR), HAS3 (siHAS3), or CD44 siRNA (siCD44) oligos for 48 h and then the efficiency of HAS3 or CD44 silencing has been measured by qRT-PCR. siRNA oligos against CD44 mRNA was purchased by Qiagen (Flexitube siRNA SI03098123). Bars represent the mean of three independent experiments (*n* = 3, biological replicates) ± SD. No data were excluded from the analysis. *p-value < 0.05. **p-value < 0.01. **b** Representative images of the mammospheres derived from HCC1954 cells transfected as in **a**. **c** HCC1954 cells transfected as in **a** were plated for the sphere-forming assay. The sphere-forming efficiency (SFE) was calculated as the percentage of the number of spheres per plated cells. Values represent the mean of the number of spheres counted in 12 (*n* = 12) different fields. The values are representative of three independent experiments (*n* = 3 biological replicates). *p-value < 0.05. **d** HCC1954 cells were plated for the sphere-forming assay in the presence of the indicated concentration of 4-MU. SFE was calculated as in **c**. Values represent the mean of the number of spheres counted in 10 (*n* = 10) different fields. The values are representative of three independent experiments (*n* = 3 biological replicates). **p-value < 0.01.

To further investigate the functional relationship between ΔNp63, HA metabolism, and breast tumor stemness, we silenced HAS3 or CD44 expression in HCC1954 cells (Fig. 3a) and then we evaluated the capacity of the silenced cells to form mammospheres in vitro. At morphological level, the depletion of HAS3 and, at higher extent, CD44 decreases the number and the size of mammospheres (Fig. 3b). To quantify this effect, we measured the sphere forming efficiency (SFE) of the silenced cells with respect to the control, and we confirmed that depletion of HAS3 or CD44 significantly decreases the ability of HCC1954 cells to form mammospheres in vitro (Fig. 3c). Similarly, the chemical inhibition of the HA synthase activity by 4-MU decreased the mammosphere forming capacity of HCC1954 cells (Fig. 3d).

Taken together, these findings indicated that the HA/CD44 pathway contributes to sustain the stemness properties of basal-type breast cancer cells.

### HAS3 is a negative prognostic factor for TNBC patient survival

**Fig. 4:**
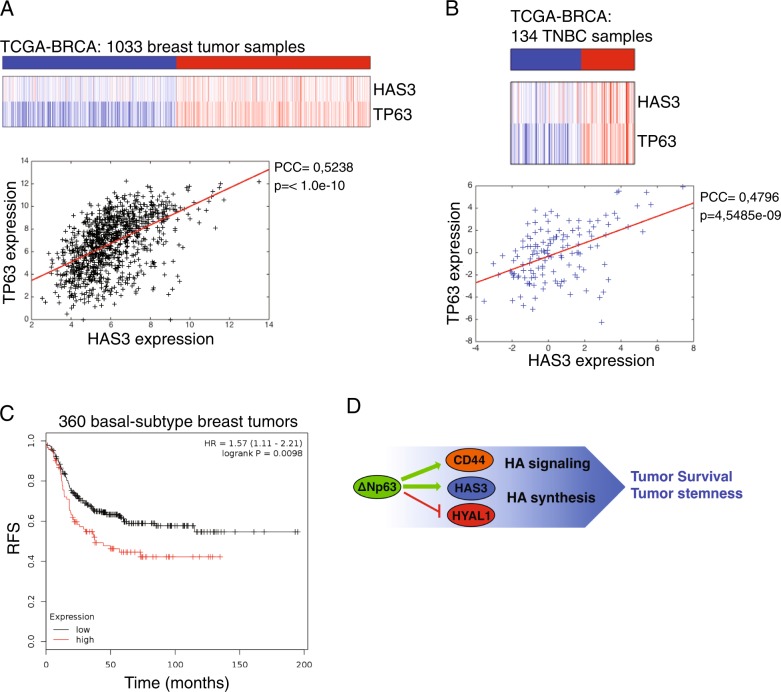
HAS3 expression is a negative prognostic factor of TNBC patient survival. Analysis of TP63 and HAS3 co-expression in breast tumors (TCGA-BRCA, 1033 patients) (**a**) and in triple-negative breast tumors (TCGA-BRCA, 134 patients) datasets (**b**). Pearson’s correlation coefficient (PCC) was calculated for primary tumors using the matlab routine corr. **c** Basal-type breast tumor samples from a cohort of 360 patients were clustered into two groups displaying low and high HAS3 expression. The lower and upper quartiles were selected by using the best performing threshold as cutoff. Relapse-free survival (RFS) has been calculated (*p*-value: 0.0068). **d** Schematic model of the ΔNp63-mediated regulation of the HA metabolism and signaling

Since ΔNp63 is able to positively regulate HAS3 expression in breast tumor cell lines, we hypothesized that HAS3 and p63 expression should be positively correlated in human breast tumors. To test this, we interrogated human breast cancer dataset comprising 1033 human breast tumor expression profiles and we found that HAS3 and p63 expression is positively correlated (PCC: 0.52; *p-*value: < 1.0e−10) (Fig. 4a). Interestingly, this correlation is also evident in 134 TNBC tissues (PCC: 0.47; *p*-value: 4.54e−09) (Fig. 4b) reinforcing the idea that p63–HAS3 axis might be functionally important in the pathobiology of TNBC. In order to test whether HAS3 expression might be a prognostic marker in breast tumors, we analyzed the relapse-free patient survival (RFS) of two groups of TNBC cancer affected patients: those displaying high or low HAS3 expression. We found that patients with high HAS3 expression show a decrease of RFS compared to those with low expression (Fig. 4c). Collectively, our data indicated that in basal-type breast tumor cells ΔNp63 is able to sustain the production of the HA by controlling the expression of the HA-related genes, and such transcriptional program might be functionally important for TNBC progression (see the schematic model in Fig. 4d).

Previous reports have demonstrated that ΔNp63 regulates a subset of cell adhesion molecules and components of ECM, such as fibronectin, collagen, and integrin receptors^54^. Our data unveil a novel pathway linking the pro-oncogenic activity of ΔNp63 with the metabolism of HA, one important component of the ECM. During normal development and tumor progression HA undergoes extensive remodeling and many human tumors are characterized by high amounts of tumor cells-associated HA^41,55,56^. Notably, high levels of HA within human tumors correlate with the malignant progression in many tumor types, including human breast cancer^57^. The role of HA in various aspects of tumors pathobiology depends not only on its levels but also on the size of HA polymers^43,58^^59^. In this report, we show that ΔNp63 positively regulates the expression HAS3, an HA synthase enzyme capable to synthesize low molecular weight chains of HA that might exert a pro-tumorigenic action^60^. Accordingly, the overexpression of HAS3 in breast cancer, osteosarcoma and colon carcinoma is associated with higher malignancy^57,61,62^. Interestingly, we noticed that the chemical inhibition of HA synthase or HAS3 silencing (see Fig. 2e and our unpublished results) decreases the expression of ΔNp63, suggesting a potential feedback regulatory loop that ensure high expression of ΔNp63 in tumor cells embedded in a HA-rich tumor microenviroment. Accordingly, we observed a positive correlation between p63 and HAS3 expression in human breast tumor tissues.

In addition to HAS3, ΔNp63 regulates the HA catabolism by repressing the expression of the hyaluronidase gene *HYAL-1*. HYAL-1 is an acid-active lysosomal enzyme that degrades the 20-kDa fragments of HA produced by the plasma membrane-associated enzyme HYAL-2^63^. Interestingly, these 20-kDa fragments of HA are highly angiogenic and pro-tumorigenic^64^. Therefore, ∆Np63 can favor the accumulation of pro-tumorigenic fragments of HA by blocking HYAL-1 expression, and, at the same time, enhance the synthesis of pro-proliferative HA chains by activating the expression of HAS3. These pathways might act in concert to allow the production of specific HA chains that can create a favorable niche for the growth and spread of malignant cells. In agreement with a pro-tumorigenic role of the ∆Np63-mediated regulation of HA metabolism, we found that in TNBC patients high HAS3 expression is a negative prognostic factor of patient survival and it might be thus functionally important to regulate tumor progression.

In addition to regulating HA metabolic genes, our data indicated that ∆Np63 directly drives the expression of CD44, the main cell surface receptor for HA. CD44 is considered as a marker of stem cells and its expression has been linked to high self-renewal and metastatic capability of breast cancer cells^47,65^. Interestingly, ΔNp63 is a crucial regulator of stemness in both normal and malignant mammary tissues. It has been showed that in basal-type and luminal-type breast carcinoma loss of ΔNp63 decreases the self-renewal ability of cancer stem cells by controlling the signaling of Hedgehog and WNt/β-catenin pathways^19,20,66^. Our data add the HA/CD44 pathway as another player in the complex signaling regulating breast tumor stemness. We found that CD44 or HAS3 silencing decreases the self-renewal ability of breast cancer stem cells in vitro. We observed a similar effect also in cells treated with the HA synthase inhibitor 4-MU, suggesting that the HA metabolism might be involved in the CD44-mediated effect on tumor stemness.

Several lines of evidence indicate that HA/CD44 complex favors the activation of several tyrosine kinase receptors, such as the EGF-R, whose signaling is important to sustain the proliferation and survival of cancer stem cells.^67^ We found that in basal-type breast carcinoma cell lines, ∆Np63 depletion or HA synthesis inhibition decreases the activation of EGF-R, suggesting that ∆Np63 might not only regulate the tumor architecture by remodeling the HA components of the ECM, but also activate in a HA-dependent manner pro-proliferative and pro-survival pathways. In conclusion, our data support the idea that the pro-tumorigenic action of ΔNp63 is intimately linked to its ability to modify the ECM in order to create a microenvironment favorable to support the growth and survival of tumor cells.

## Electronic supplementary material

Figure S1

Figure S2

SI text
