# Supplementary figures and images for: ΔNp63 regulates the expression of hyaluronic acid-related genes in breast cancer cells
Source: Oncogenesis. 2018 Aug 24;7(8):65. doi: 10.1038/s41389-018-0073-3 (PMC6107578; doi:10.1038/s41389-018-0073-3)

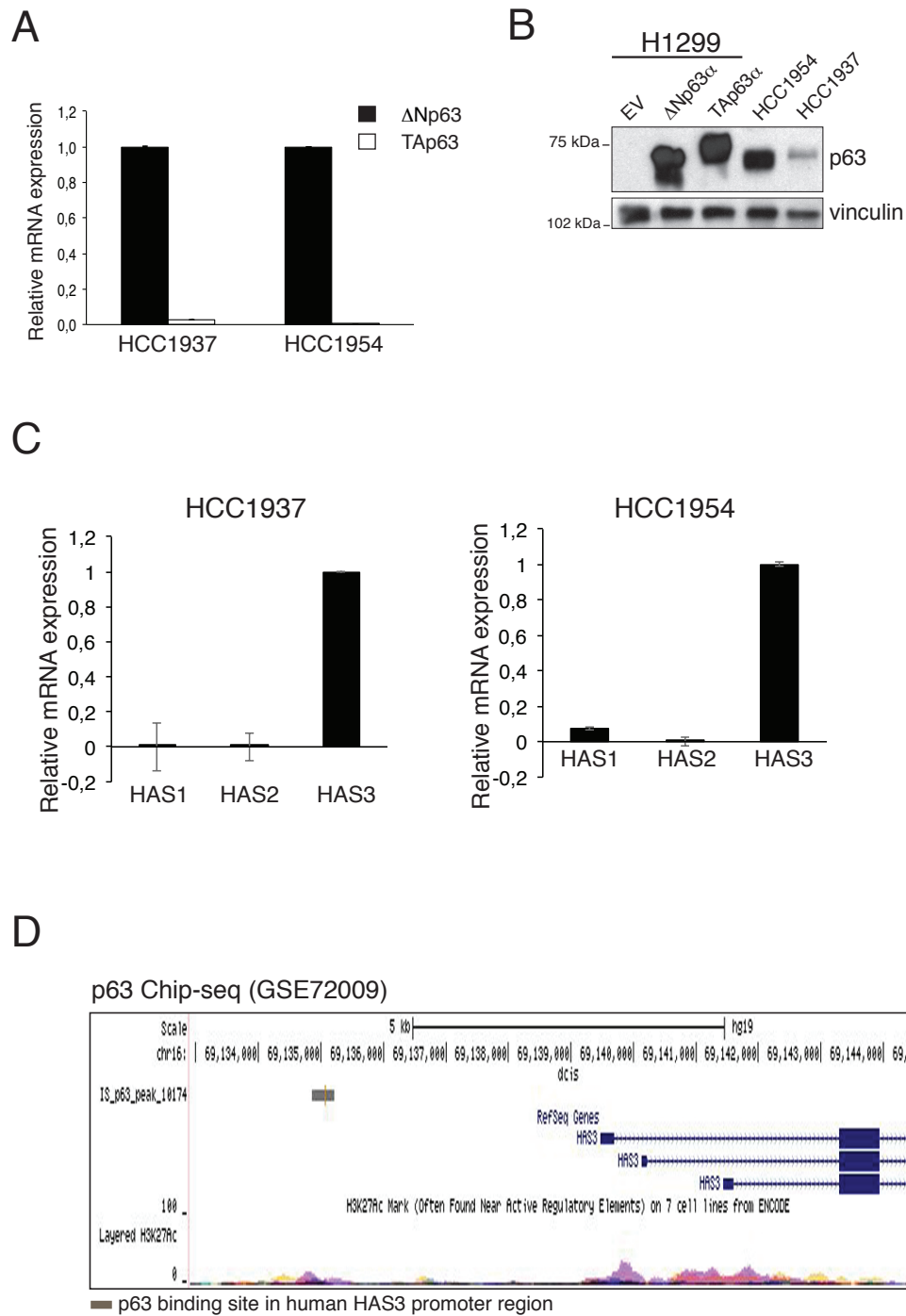

Figure S1

Supplement: Supplementary file 1 — Figure S1 [file 41389_2018_73_MOESM1_ESM.pdf]

# p63 Chip-seq (GSE72009)

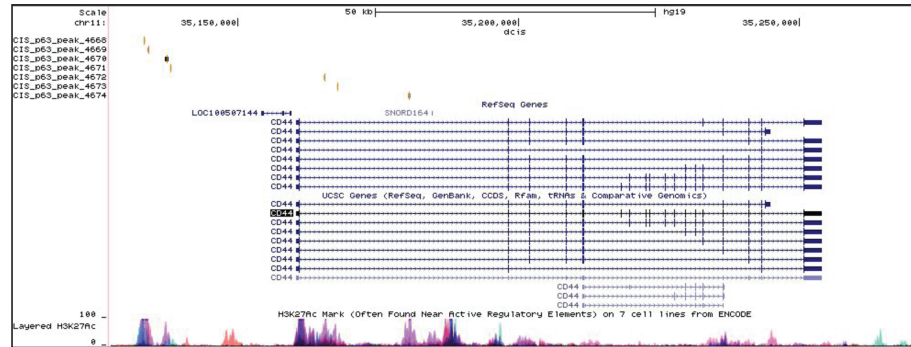

! p63 binding site in human CD44 genomic locus

Figure S2

Supplement: Supplementary file 2 — Figure S2 [file 41389_2018_73_MOESM2_ESM.pdf]
