# Supplementary material for: ΔNp63 regulates the expression of hyaluronic acid-related genes in breast cancer cells
Source: Oncogenesis. 2018 Aug 24;7(8):65. doi: 10.1038/s41389-018-0073-3 (PMC6107578; doi:10.1038/s41389-018-0073-3)
Supplement: Supplementary file 3 — SI text [file 41389_2018_73_MOESM3_ESM.pdf]

Supplementary Figure Legends

ΔNp63 regulates the expression of hyaluronic acid-related genes in breast cancer cells

Veronica Gatti, Claudia Fierro, Mirco Compagnone, Federica Giangrazi, Elke Katrin Markert, Lucilla Bongiorno-Borbone, Gerry Melino and Angelo Peschiaroli

Figure S1. (A) Analysis of TAp63 and ΔNp63 expression in the basal-type breast carcinoma cell lines HCC1937 and HCC1954. TAp63 and ΔNp63 mRNA levels were measured by qRT-PCR. The following primers were utilized: human TAp63 for 5'-TCAGAAGATCGTGC GACAAAC-3'; rev 5'-GTTCAGGAGCCCCAGGTTCG-3'; human ΔNp63 for 5'-GAAGAAAGGACAGCA GCATTG-3'; human Actin for 5'-GTTGCTATCCAGGCTG TG-3'; rev 5'-AATGTCACGCAC GATTTCCCG-3'. Bars represent the mean of three technical replicates (n=3, PCR runs) ± SD. No data were excluded form the analysis. (B) Immunoblotting (IB) analysis of p63 protein levels in the HCC1937 and HCC1954 breast tumor cell lines. As control of p63 isoforms molecular weight, protein extracts of H1299 ectopically expressed ΔNp63α or TAp63α isoforms were utilized. H1299 cells (non-smal cell lung cancer cell line) were cultured in Dulbecco's modified Eagle's medium (Gibco, Invitrogen) supplemented with 10% v/v fetal bovine serum (FBS), 100 µg/ml penicillin and 100 µg/ml streptomycin (Gibco, Invitrogen). Cells were cultured at 37 °C with 5% CO_2 and routinely tested for mycoplasma contaminations. C) HAS1, HAS2 and HAS3 mRNA levels were quantified by qRT-PCR in the indicated basal-type breast tumor cell lines. The primers used for qRT-PCR are the following: human HAS1 for 5'-TCCTGGGTCAGCTTCCTAAGC-3'; rev 5'-GACCGCTGATGCAGGAT ACA-3'; human HAS2 for 5'-TTCAGCGCACCTGTGCAT-3'; rev 5'-AA GCAGCTGTGATTCCA AGGA-3'; Bars represent the mean of three technical replicates (n=3, PCR runs) ± SD. No data were excluded form the analysis. (D) Analysis of the p63 Chip-seq data (GEO accession: GSE72009) obtained in the p63 expressing basal-subtype breast cancer cell line, MCFDCIS. p63 DNA-binding site (BS) in the human HAS3 locus (HAS3 gene is shown from left to right) is shown.

Figure S2. Analysis of the p63 Chip-seq data (GEO accession: GSE72009) obtained in the p63 expressing basal-subtype breast cancer cell line, MCFDCIS. Multiple p63 DNA-binding site in the human CD44 locus (CD44 gene is shown from left to right) are shown.
